# Reconstruction of dermal layers evaluated by high-frequency ultrasound following treatment for skin fibrosis

**DOI:** 10.3205/000318

**Published:** 2023-05-26

**Authors:** Lívia Maria Pereira de Godoy, Jose Maria Pereira de Godoy

**Affiliations:** 1 Instituto Lauro de Souza Lima, Bauru, Brazil; 2 Clínica Godoy, São José do Rio Preto, Brazil; 3 Departamento de Cardiologia e Cirurgia Cardiovascular, Faculdade de Medicina de São José do Rio Preto (FAMERP), São José do Rio Preto, Brazil; 4 Conselho Nacional de Desenvolvimento Científico e Tecnológico (CNPq), Brazil

**Keywords:** reconstruction of dermal layers, high-frequency ultrasound, treatment for skin fibrosis

## Abstract

**Background:**

Lymphedema is a chronic, progressive clinical condition that evolves with intense fibrosis, the most advanced stage of which is stage III (lymphostatic fibrosclerosis).

**Aim:**

The aim of the present study was to show the possibility to reconstruct the dermal layers with the intensive treatment of fibrosis using the Godoy method.

**Case description:**

A 55-year-old patient with an eight-year history of edema of the lower limb of the leg had constant episodes of erysipelas, despite regular treatments. The edema progressed continually, associated with a change in the color of the skin and the formation of a crust. Intensive treatment (eight hours per day for three weeks) was proposed with the Godoy method. The ultrasound was performed and results revealed substantial improvement in the skin, with the onset of the reconstruction of the dermal layers.

**Conclusion:**

It is possible to reconstruct the layers of the skin in fibrotic conditions caused by lymphedema.

## Zusammenfassung

**Hintergrund:**

Das Lymphödem ist ein chronischer, fortschreitender klinischer Zustand, der sich mit einer starken Fibrose entwickelt, deren fortgeschrittenstes Stadium das Stadium III (lymphatische Fibrosklerose) ist.

**Ziel:**

Ziel der vorliegenden Studie war es, die Möglichkeit der Rekonstruktion der Hautschichten bei intensiver Behandlung der Fibrose nach der Godoy-Methode aufzuzeigen.

**Fallbericht:**

Ein 55-jähriger Patient, der seit acht Jahren an einem Ödem der unteren Extremität litt, hatte trotz regelmäßiger Behandlung immer wieder Schübe von Erysipel. Das Ödem schritt kontinuierlich voran, verbunden mit einer Veränderung der Hautfarbe und der Bildung einer Kruste. Eine intensive Behandlung (acht Stunden pro Tag für drei Wochen) nach der Godoy-Methode wurde vorgeschlagen. Wir haben eine Ultraschalluntersuchung durchgeführt, deren Ergebnisse eine deutliche Verbesserung der Haut und den Beginn des Wiederaufbaus der Hautschichten zeigten.

**Schlussfolgerung:**

Es ist möglich, die Hautschichten bei fibrotischen Zuständen, die durch Lymphödeme verursacht werden, zu rekonstruieren.

## Introduction

Lymphedema is a chronic, progressive, clinical condition that evolves with intense fibrosis, the most advanced stage of which is stage III (lymphostatic fibrosclerosis) [1], [2]. In more developed countries, lower limb lymphedema may be primary (congenital) or secondary to the surgical and radiotherapeutic treatments of tumors. In countries such as India this condition is mainly secondary to filariasis, but other infectious causes are frequent [1], [2], [3]. With regards to treatment, a considerable advance emerged with novel concepts and forms of treatment developed by Godoy & Godoy, which enable approximately a 50% reduction in the volume of the affected limb after five days of intensive treatment and the eventual normalization or near normalization in all clinical stages, including lymphostatic fibrosclerosis [4], [5], [6].

High-frequency ultrasound enables viewing the epidermis, dermis and hypodermis, the precise measurement of the thickness of the skin as well as the assessment of edema, fibrosis and atrophy of the skin [7]. One study reports that this imaging method can precisely detect lymphatic vessels for efficient lymphatic microsurgery without the prior need for lymphangiography [8]. A noninvasive, quantitative comparison of dermal fibrosis using optical coherence tomography (OCT) and high-frequency ultrasound (HFUS) revealed that both methods achieve the precise measurement of structural and physiological changes in the skin, but epidermal and dermal structures are better distinguished using OCT [8].

There are no studies in the literature showing the restoration of the dermis in patients with skin fibrosis. The aim of the present study was to show the possibility of the restoration of the dermal layers with the intensive treatment of fibrosis using the Godoy method.

## Case description

**Figure 1:**
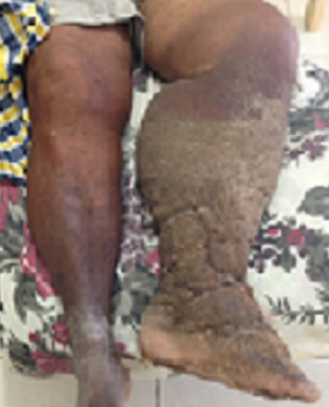
Initial treatment

**Figure 2:**
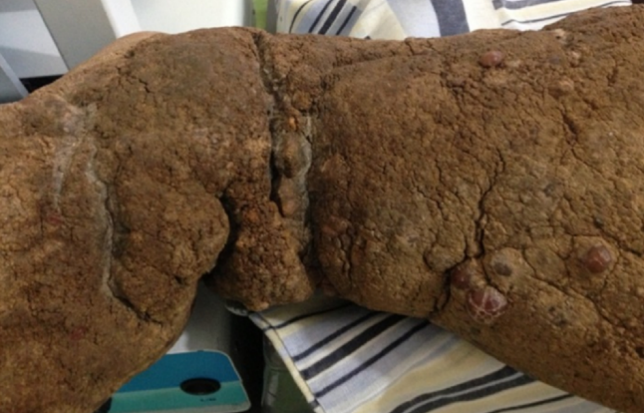
Skin with an intense formation of crust

A 55-year-old patient with an eight-year history of edema of the lower limb of the leg had constant episodes of erysipelas, despite regular treatments (Figure 1 (Fig. 1)). The edema progressed continually, associated with a change in the color of the skin and the formation of a crust (Figure 2 (Fig. 2)). However, the patient underwent no specific treatment for lymphedema until obtaining access to the Clínica Godoy-Brazil. The physical examination revealed lymphedema that limited the mobility of the ankle and a hardened crust below the knee (Figure 2 (Fig. 2)).

Intensive treatment (eight hours per day for three weeks) was proposed with the Godoy method. Treatment involved mechanical lymphatic therapy using the RAGodoy^®^ device eight hours per day and cervical lymphatic stimulation using the Godoy and Godoy technique 15 minutes per day. The skin was protected with an Unna boot. A hand-crafted non-elastic stocking made with grosgrain fabric was placed over the affected limb. The stocking was crafted to fit the measurements of the limb and adjusted based on the reduction in volume.

**Figure 3:**
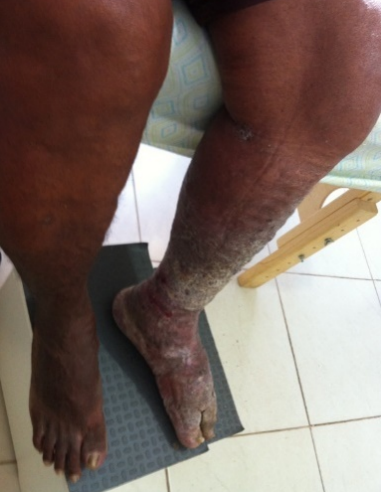
After treatment

**Figure 4:**
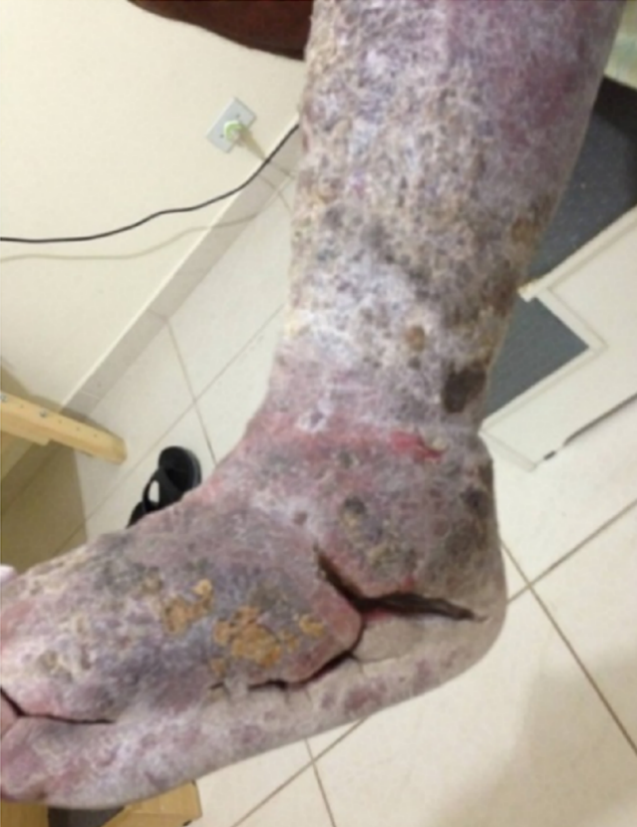
Considerable peeling of the skin, which remains dry and dehydrated despite constant moisturizing

**Figure 5:**
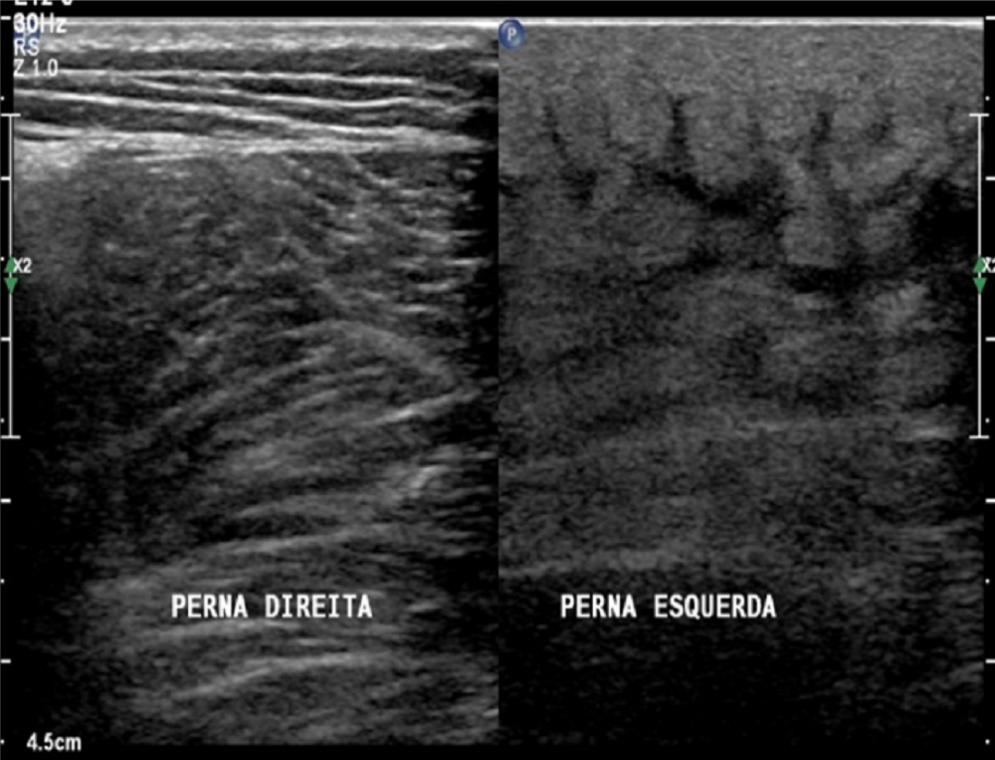
High-frequency ultrasound. Left side of the image: normal right lower limb; right side of the image: left lower limb with lymphedema and disarrangement of skin structures

**Figure 6:**
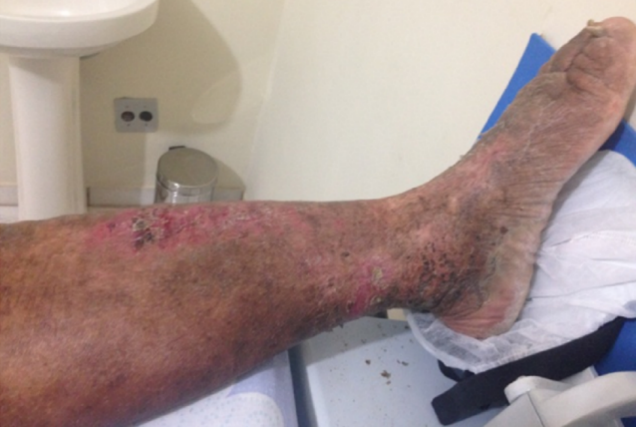
Skin with substantial improvement, achieving near clinical normality

**Figure 7:**
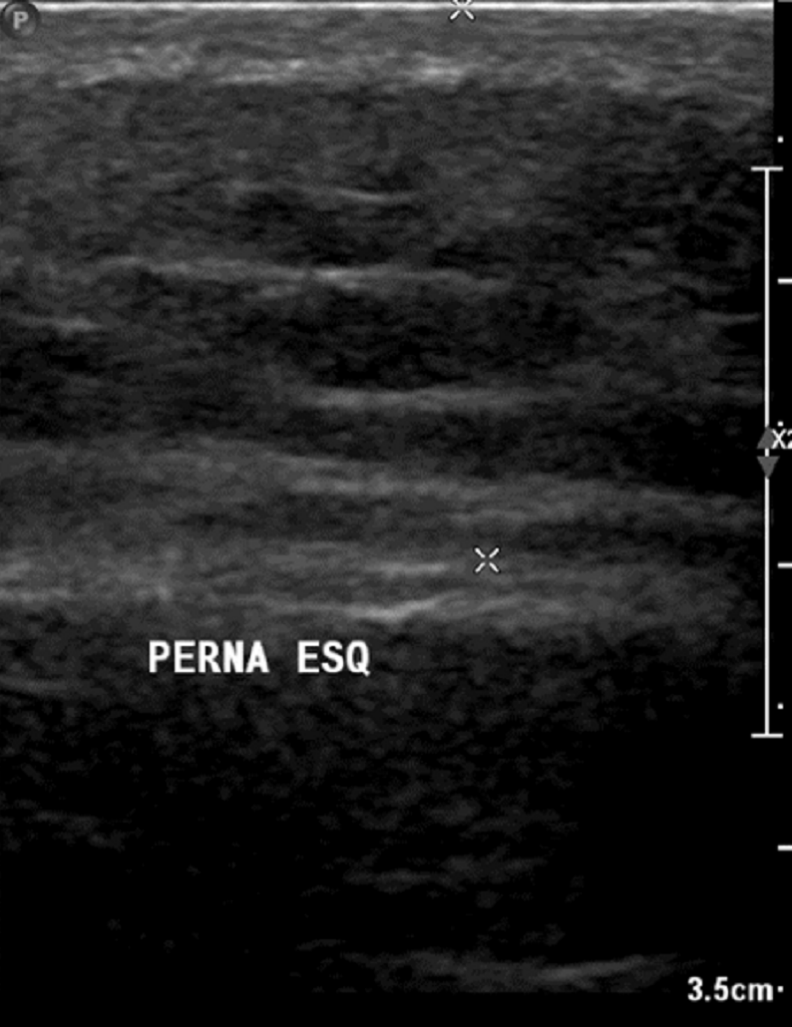
Post-treatment ultrasound showing the onset of the reconstruction of the skin layers

The reduction in volume of the limb was constant with treatment (Figure 3 (Fig. 3)). The skin received moisturizing and peeling occurred (Figure 4 (Fig. 4)). At this point, HFUS was performed, which revealed the disarrangement of the structures of the dermal layers and intensive fibrosis (Figure 5 (Fig. 5)). Treatment led to considerable improvement in the skin to standards close to normality (Figure 6 (Fig. 6)) and a new ultrasound was performed (Figure 7 (Fig. 7)). The results revealed substantial improvement in the skin, with the onset of the restoration of the dermal layers. At this point, the patient began to undergo outpatient treatment, maintaining the moisturizing of the skin and use of the grosgrain stocking.

## Discussion

The present study described the reversal of skin fibrosis to nearly clinical normality and the restoration of the skin layers following intensive lymphatic treatment using the Godoy method in a patient with lymphedema that led to the formation of an intense crust. No studies in the literature describe the use of this therapeutic strategy or show similar results.

The clinical reversal of fibrosis is a constant finding in the treatment of lymphedema, as the intensive Godoy method enables approximately a 50% reduction in the volume of the affected limb in five days of treatment, achieving a 70 to 90% reduction in volume in the second week [4], [5], [6]. The intensity of such reductions depends on the volume of the limb and the fibrotic process. However, the aim is to achieve normality or near normality, as described in the present case study.

The first phase of treatment is to achieve the clinical reversal of fibrosis associated with lymphedema in all clinical stages, including stage lymphostatic fibrosclerosis. In this phase, we are seeking to assess the structural changes of the skin in the fibrotic process and its reversal. High-frequency ultrasound is a noninvasive exam with good definition that allows to determine whether important changes have occurred in the skin layers [9].

As a noninvasive exam, high-frequency ultrasound is an important tool for the assessment of the dermis in these patients during treatment. A comparison with the results of the biopsies constitutes the most recent phase of our study [10], [11]. However, the results demonstrated the possibility of the reversal of fibrosis in patients with more advanced phases of lymphedema.

Another important detail in this case was the resolution of the intense crust on the patient’s leg. Therefore, this study offers a line of investigation to detect the structures composing the skin that interfere with these changes. Biopsy is fundamental to gaining a better understanding of why this patient had such considerable crust formation.

## Conclusion

It is possible to reconstruct the layers of the skin in fibrotic conditions caused by lymphedema.

## Notes

### Authors’ ORCIDs

- Lívia Maria Pereira de Godoy: 0000-0002-1036-8779
- Jose Maria Pereira de Godoy: 0000-0001-5424-7787

### Acknowledgment

The authors would like to acknowledge Dr. Miriam Tarraf Fernandes and Dr. Julio Roberto Fernandes for their contribution to the performance and interpretation of ultrasound assessments.

### Ethics approval

The study was approved by the Ethical Committee of Faculdade de Medicina de Sao Jose do Rio Preto-FAMERP-Brazil # 4.397.868. 

### Competing interests

The authors declare that they have no competing interests.
